# Motion‐compensated b‐tensor encoding for in vivo cardiac diffusion‐weighted imaging

**DOI:** 10.1002/nbm.4213

**Published:** 2019-11-25

**Authors:** Samo Lasič, Filip Szczepankiewicz, Erica Dall'Armellina, Arka Das, Christopher Kelly, Sven Plein, Jürgen E. Schneider, Markus Nilsson, Irvin Teh

**Affiliations:** ^1^ Random Walk Imaging Lund Sweden; ^2^ Clinical Sciences Lund University Lund Sweden; ^3^ Harvard Medical School Boston Massachusetts USA; ^4^ Brigham and Women's Hospital Boston Massachusetts USA; ^5^ Leeds Institute of Cardiovascular and Metabolic Medicine University of Leeds Leeds UK; ^6^ Division of Cardiovascular Medicine, Radcliffe Department of Medicine University of Oxford Oxford UK

**Keywords:** acceleration nulling, b‐tensor encoding, cardiac MRI, concomitant field, isotropic diffusion weighting, motion compensation, velocity nulling

## Abstract

Motion is a major confound in diffusion‐weighted imaging (DWI) in the body, and it is a common cause of image artefacts. The effects are particularly severe in cardiac applications, due to the nonrigid cyclical deformation of the myocardium. Spin echo‐based DWI commonly employs gradient moment‐nulling techniques to desensitise the acquisition to velocity and acceleration, ie, nulling gradient moments up to the 2nd order (M2‐nulled). However, current M2‐nulled DWI scans are limited to encode diffusion along a single direction at a time.

We propose a method for designing b‐tensors of arbitrary shapes, including planar, spherical, prolate and oblate tensors, while nulling gradient moments up to the 2nd order and beyond. The design strategy comprises initialising the diffusion encoding gradients in two encoding blocks about the refocusing pulse, followed by appropriate scaling and rotation, which further enables nulling undesired effects of concomitant gradients. Proof‐of‐concept assessment of in vivo mean diffusivity (MD) was performed using linear and spherical tensor encoding (LTE and STE, respectively) in the hearts of five healthy volunteers. The results of the M2‐nulled STE showed that (a) the sequence was robust to cardiac motion, and (b) MD was higher than that acquired using standard M2‐nulled LTE, where diffusion‐weighting was applied in three orthogonal directions, which may be attributed to the presence of restricted diffusion and microscopic diffusion anisotropy.

Provided adequate signal‐to‐noise ratio, STE could significantly shorten estimation of MD compared with the conventional LTE approach. Importantly, our theoretical analysis and the proposed gradient waveform design may be useful in microstructure imaging beyond diffusion tensor imaging where the effects of motion must be suppressed.

## Graphical abstract

Diffusion MRI in the heart is confounded by motion and long acquisition times. A novel design strategy for diffusion encoding is proposed to generate q‐trajectories corresponding to b‐tensors of arbitrary shapes, with gradient moment nulling up to arbitrary order and compensation for concomitant gradient effects. We demonstrate proof‐of‐concept application of acceleration‐compensated isotropic diffusion encoding (q‐trajectory shown in figure) for rapid in vivo mean diffusivity measurement in the human heart.

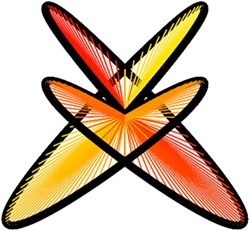

**Abbreviations used**
DW: diffusion‐weighted
DWI: diffusion‐weighted imaging
LTE: linear tensor encoding
LTEmin: linear tensor encoding with minimised echo time
LV: left ventricular
MD: mean diffusivity
PNS: peripheral nerve stimulation
RF: radio frequency
SD: standard deviation
SE: spin echo
SNR: signal‐to‐noise‐ratio
STE: spherical tensor encoding

## 1 INTRODUCTION

Diffusion‐weighted imaging (DWI) is highly susceptible to bulk motion artefacts due to physiological processes such as peristalsis, respiration and cardiac pulsation. Of the many body applications that are affected by motion, including liver, kidney and prostate, one of the most challenging applications is imaging of the heart.

To overcome the confounds of bulk tissue motion, two main approaches have been adopted. The first is a cardiac‐triggered stimulated echo acquisition mode (STEAM) echo planar imaging (EPI) method^1^ that utilises monopolar gradient waveforms applied across two consecutive cardiac cycles. The long diffusion times of the order of 1 second mean that high b‐values can be achieved with relatively modest gradient hardware. In addition, DWI data can be acquired over much of the cardiac cycle, facilitating dynamic assessment of microstructural changes.^2^ However, STEAM suffers from a loss of half the signal,^3^ sensitivity to myocardial strain,^4^ and the need for breath‐holding over extended periods to minimise respiratory motion. These limitations can be largely overcome using motion‐compensated spin‐echo (SE) DWI. Initial SE methods with gradient moments nulled up to the 1st order (M1‐nulling), ie, bulk velocity‐nulling,^5^ were superseded by protocols even more robust to bulk motion with gradient moments nulled up to the 2nd order (M2‐nulling), ie, with both velocity and acceleration nulled.^6^ Nulling moments up to the 3rd order, ie, jerk‐nulling, was explored in rat hearts on a preclinical scanner,^7^ but the longer echo times led to lower signal‐to‐noise ratio (SNR), and M2‐nulled methods remain the mainstay of SE DWI. These waveforms were applied in diffusion‐prepared fast spin echo^8^ and balanced steady‐state free precession readouts,^9^ but more often in single‐shot SE EPI sequences. Compared with STEAM, SE methods yield higher SNR,^10^ and data can be acquired while free‐breathing, improving their applicability in patient cohorts. However, SE methods require comparatively stronger gradient systems,^11^ and are less robust outside the mid‐systolic phase of the cardiac cycle.^12^ If gradient waveforms are allowed to be asymmetric with respect to the refocusing pulse in SE DWI, M2‐nulled encoding can be optimised with the benefit of shorter TE and higher SNR.^13^ However, if not accounted for, asymmetric waveforms are prone to undesired effects of concomitant gradients leading to signal bias and image artefacts.^14^

To date, gradient waveform development in cardiac DWI has focused on diffusion encoding along a single direction at a time, relying on multiple acquisitions with encoding in different directions, for example, in diffusion tensor imaging (DTI),^15^ allowing the obtaining of rotationally invariant measures. This can be time‐consuming, particularly in conjunction with cardiac triggering that is routinely used. When only mean diffusivity (MD) is of interest, isotropic diffusion weighting can be applied.^16^, ^17^, ^18^, ^19^ MD can thus in principle be obtained with two acquisitions rather than four, with the potential of accelerating the measurement at least 2‐fold.^17^, ^20^, ^21^ In applications such as cardiac MRI, it is often desirable to minimise the contribution of perfusion, which would otherwise lead to an overestimation of MD by acquiring nonzero low b‐values instead of non‐DW data.^10^, ^22^ In this case, at least six acquisitions would be required, thus the potential acceleration is 3‐fold. To our knowledge, isotropic diffusion weighting has only been implemented with nulling of gradient moments up to the 1st order.^16^

Further limitations of conventional diffusion encoding applied along a single direction arise in the context of microstructure studies of heterogeneous materials. It has been realised that more specific microstructural information can only be assessed if the encoding direction is varied during the diffusion weighting interval. At first, experiments employed encoding along two orthogonal directions.^23^ Eriksson et al introduced isotropic diffusion weighting by magic angle spinning of the q vector^19^ as a means to probe microscopic diffusion anisotropy unconfounded by orientation dispersion, which is caused, for example, by elongated cells.^24^, ^25^ The methodology was later generalised, and the concept of encoding with b‐tensors was introduced^19^, ^26^, ^27^ (also referred to as tensor‐valued diffusion encoding^14^, ^28^). These waveforms can be numerically optimised, while accounting for practical constraints such as gradient strength, slew rate and heating,^29^ and permitted clinically applicable protocols with modest scan times and gradient hardware requirements.^30^ Szczepankiewicz et al^14^ have analysed the effects of concomitant fields in tensor‐valued encoding and have suggested a gradient waveform design that minimises these effects at the encoding stage.

In this study, we propose a novel gradient waveform design for tensor‐valued diffusion encoding with nulling of motion up to arbitrary orders. Importantly, our strategy includes nulling the effects of concomitant field gradients. We show proof‐of‐concept in vivo application in the hearts of healthy volunteers and evaluate the performance of M2‐nulled isotropic encoding and its potential for rapid MD measurement.

## 2 THEORY

### 2.1 Desired properties of encoding

Consider a SE radio frequency (RF) pulse sequence with an effective diffusion encoding gradient vector **g**(*t*), which accounts for the spin‐dephasing direction after application of the refocusing RF pulse. At the end of the encoding interval (*τ*_E_), the effects of coherent spin motion can be eliminated by nulling gradient moments, **m**_*n*_(*τ*_E_) = 0, where

(1) $$m_{n}\left(t\right)=\int_{0}^{t}g\left(u\right)u^{n}\mathrm{du}$$

and *n* is the moment order. When **m**_*n*_(*τ*_E_) = 0, the SE signal will not be sensitive to the *n*th time derivative of spin‐position, eg, velocity, acceleration, jerk for *n* = 1, 2, 3. This is generally valid only when the velocity, acceleration, jerk, etc. are constant over the diffusion encoding period. The SE condition requires that **m**_0_(*τ*_E_) = 0.

The spin‐dephasing trajectory, also known as the q‐trajectory,^28^ is given by

(2) $$q\left(t\right)=\gamma \;m_{0}\left(t\right),$$

yielding the diffusion encoding tensor as^26^, ^27^, ^28^

(3) $$b=\gamma^{2}\int_{0}^{\tau_{E}}m_{0}\left(u\right)⨂m_{0}\left(u\right)\mathrm{du},$$

where ⨂ represents tensor product and *γ* is the gyromagnetic ratio. The b‐value is given by the trace of b‐tensor, *b* = tr(**b**). We refer to tensor rank as the number of nonzero eigenvalues. The conventional encoding is described by rank 1 b‐tensors and is often termed linear tensor encoding (LTE). By contrast, isotropic diffusion weighting is described by spherical b‐tensors, ie, a special case of rank 3 tensors, thus termed spherical tensor encoding (STE). If a linear transformation (scaling and rotation), **L**, is applied to a gradient vector **g**′, so that **g = Lg**′, then **q = Lq**′ and **b = Lb** ′ **L**^**T**^**.** 28 Linear transformations can be applied to an initial encoding scheme **g**′ to yield b‐tensors of different sizes and shapes.

### 2.2 General strategy for nulling gradient moments

**Figure 1:**
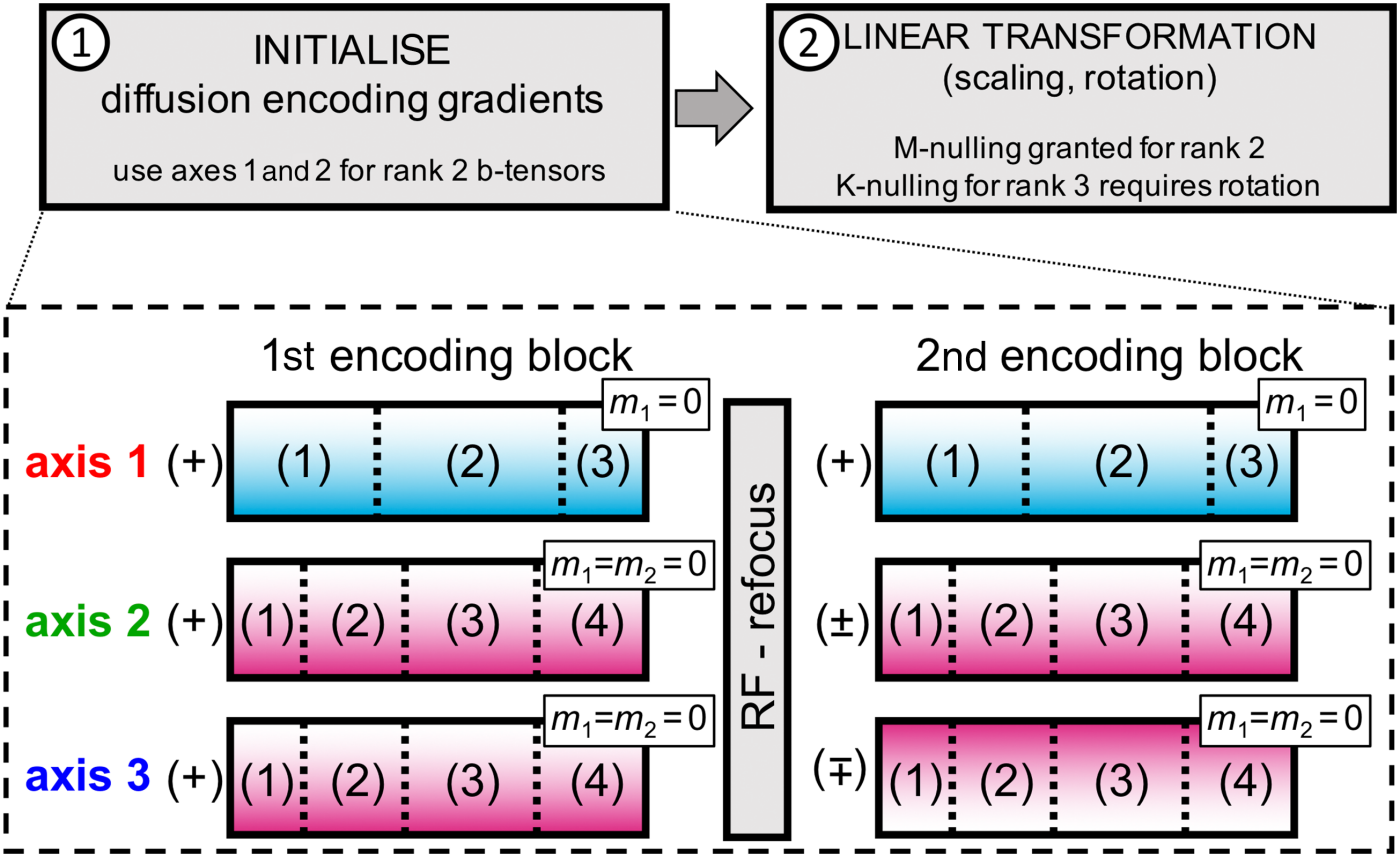
schematic for designing a spin‐echo (SE) sequence that yields tensor‐valued diffusion encoding with gradient moment nulling up to 2nd order as well as nulling effects of concomitant gradients. Encoding is designed in two steps: (i) gradient waveforms are initialised, and (ii) a linear transformation (scaling, rotation) is applied to achieve desired b‐tensor shape and size and to null elements of tensor **K**. the schematic within the dashed frame illustrates the initialisation step. Note that in this step the axis ordering is arbitrary. Encoding is separated into two encoding blocks of equal duration before and after refocusing pulse. Within a single encoding block, each gradient waveform is divided into three (axis 1) or four (axes 2 and 3) subintervals of arbitrary durations (note the unevenly positioned vertical dashed lines) for nulling gradient moments up to 1st or 2nd order (*m*_1_ = 0 or *m*_1_ = *m*_2_ = 0), respectively. The two within‐block gradient waveforms are copied with equal or opposite polarities across axes and encoding blocks. The polarities (+) or (−) here refer to gradients in the laboratory system (not effective gradients). The same polarity (+) is used for the two encoding blocks along axis 1 to null gradient moments up to 2nd order at the end of encoding. Polarity needs to change between the two encoding blocks along either one of the axes 2 or 3 to yield rank 3 b‐tensors. For rank 2 b‐tensors, gradients along either axis 2 or axis 3 can be set to zero. However, to null effects of concomitant gradients independent of rotation in rank 2 b‐tensor encoding, equal polarities for the two encoding blocks need to be used

Let us first consider gradient moments along a single encoding axis. Further information related to our pulse sequence design (Figure 1) for generating motion‐compensated tensor‐valued encoding is provided in the Methods section. The entire encoding duration *τ*_E_ can be considered as a single encoding block or a concatenation of several encoding blocks. Here, an encoding block refers to an encoding interval during which no RF pulses are applied. A sequence with single SE thus comprises two encoding blocks. We can further divide each encoding block into *ν* subintervals of arbitrary durations within which the effective gradient *g*(*t*) does not change polarity. For a single encoding block, the effective gradient is piecewise defined in each subinterval *I*_*i*_ as $C_{i}{\tilde{f}}_{i}\left(t\right)$, where *t* ∈ *I*_*i*_, *C*_*i*_ are real constants and ${\tilde{f}}_{i}\left(t\right)$ are normalised waveforms, $\left|{\tilde{f}}_{i}\left(t\right)\right|\leq 1$. We will refer to these as gradient “lobes”. In each subinterval the accumulated gradient moment is

(4) $${∆m}_{\mathrm{ni}}=C_{i}∆{\tilde{m}}_{\mathrm{ni}},$$

where

(5) $$∆{\tilde{m}}_{\mathrm{ni}}=\int_{t\in I_{i}}{\tilde{f}}_{i}\left(t\right)t^{n}\mathrm{dt}.$$

The total accumulated gradient moment within an encoding block is nulled when

(6) $${∆m}_{n}=\sum_{i=1}^{\nu }C_{i}∆{\tilde{m}}_{\mathrm{ni}}=0.$$

To fulfil *∆m*_*n*_ = 0 for all moments up to order *n*, we have *ν* adjustable parameters given a set of waveforms ${\tilde{f}}_{i}\left(t\right)$. This requirement can be formulated as a system of linear equations^31^ by

(7) $$\tilde{m}C=0,$$

where $\tilde{m}$ is a *n* + 1 by *ν* matrix with elements $∆{\tilde{m}}_{\mathrm{ij}}$ (0 ≤ *i* ≤ *n*, 1 ≤ *j* ≤ *ν*) and **C** = (*C*_1_, …, *C*_*ν*_)^T^. Considering that the entire waveform within an encoding block can be arbitrarily scaled, we can set, eg, *C*_1_ = 1. Equation 7 thus has *ν* − 1 free parameters. The critical case to solve for **C** is when *ν* = *n*+2 *,* which sets the minimum number of encoding block subdivisions, ie, the number of gradient lobes. For velocity compensation, *n* = 1 and thus *ν* = 3. For velocity and acceleration compensation, *n* = 2 and *ν* = 4. The critical case for solving Equation 7 is implicit also in the binomial expansion approach^31^ and applies often in several other motion‐compensated diffusion encoding designs.^7^, ^32^ By contrast, an overdetermined system can be used to minimise echo times.^13^

Let us now consider two encoding blocks of equal durations, *τ*_B_, which start at times *t*_B1_ and *t*_B2_ = *t*_B1_+δ, respectively. How can residual moments after the first encoding block be nulled by the second encoding block? To address this question, we first consider how time translation of the entire encoding block affects the residual moment. Shifting the onset of an encoding block by δ and using Equation 1 yields

(8) $${∆m}_{n}\left(\delta \right)=\int_{\delta }^{\delta +\tau_{B}}g\left(t\right)t^{n}\mathrm{dt}=\int_{0}^{\tau_{B}}g\left(\delta +t\right){\left[\delta +t\right]}^{n}\mathrm{dt}.$$

By expanding [δ+*t*]^*n*^ as

(9) δ+tn=∑m=0nnmδmtn−m,

where nm=n!m!n−m! are binomial coefficients, we get

(10) ∆mnδ=∑m=0nnmδm∆mn−m0,

where the terms *∆m*_*k*_(0) with 0 ≤ *k* ≤ *n* represent accumulated moments for the nonshifted block according to Equation 8 with δ = 0. We see that when *∆m*_*k*_(0) = 0 for all *k* < *n*, *∆m*_*n*_(δ) = *∆m*_*n*_(0). This invariance of the residual moment to time translation is fundamental in the elegant gradient moment nulling strategy suggested by Pipe and Chenevert.^33^ Under the condition that all gradient moments of an order lower than *n* are nulled in the first encoding block, a copy of the first block with inverted polarity can be applied at any later time to null the moment of order *n*. We use this principle in our b‐tensor encoding design (Figure 1).

It is worth noting that it is sufficient to ensure gradient moment nulling along any three orthogonal axes. If **m**_*n*_(*τ*_E_) = 0, projection along any direction **u** is also nulled, ie, **m**_*n*_(*τ*_E_) · **u** = 0. Furthermore, any linear transformation of the gradient **Lg**(*t*) will also yield nulled moments **Lm**_*n*_(τ_E_) = 0. This is instrumental for generalisations of motion nulling in b‐tensor encoding.

### 2.3 Nulling effects of concomitant gradients

Due to the nature of magnetic fields, as described by Maxwell's equations, our intended encoding gradients are expected to cause additional undesired concomitant field gradients, which depend also on the magnitude and direction of external magnetic field density **B**_0_ and the distance from its isocentre. We will consider specific constraints due to concomitant gradients in the following sections.

To the first order, the effects of concomitant gradients, proportional to k‐space shift, are nulled when

(11) $$K\left(\tau_{E}\right)=0,$$

where

(12) $$K\left(t\right)=\frac{\gamma }{2\pi }\int_{0}^{t}h\left(u\right)G_{C}\left(u\right)du.$$

Here, **G**_C_(*t*) is the concomitant gradient matrix and *h*(*t*) assumes values of 1 or − 1, alternating sign after application of a refocusing pulse. If **B**_0_ at the isocentre is aligned along the z‐axis, the concomitant gradient matrix is given by^14^, ^34^, ^35^

(13) $$G_{C}\left(t\right)=\frac{1}{4B_{0}}\left[\begin{array}{ccc} g_{z}^{2}\left(t\right) & 0 & -2g_{x}\left(t\right)g_{z}\left(t\right)\\ 0 & g_{z}^{2}\left(t\right) & -2g_{y}\left(t\right)g_{z}\left(t\right)\\ -2g_{x}\left(t\right)g_{z}\left(t\right) & -2g_{y}\left(t\right)g_{z}\left(t\right) & 4g_{x}^{2}\left(t\right)+4g_{y}^{2}\left(t\right) \end{array}\right].$$

Note that there are four independent and nonzero elements of **G**_C_ and **K**. We will recall the condition of Equation 11 as K‐nulling.^14^ Under the symmetry conditions, which we will discuss later, K‐nulling can be fulfilled for any b‐tensor encoding waveforms, provided that the gradients are appropriately rotated relative to the main magnetic field vector **B**_0_. However, to null the effects of concomitant gradients for an arbitrary rotation of diffusion encoding gradient waveforms, a more general condition is required, formalised as^14^

(14) $$M_{\mathrm{ij}}=\int_{0}^{\tau_{E}}h\left(t\right)g_{i}\left(t\right)g_{j}\left(t\right)dt=0,\forall i,j\in \left(x,y,z\right).$$

To distinguish it from K‐nulling, we refer to the above condition as M‐nulling. Note that M‐nulling implies K‐nulling and not vice versa. M‐nulling is required only when several different rotations of encoding waveforms are needed, eg, when anisotropic rank 3 b‐tensors are used to probe microscopic anisotropy. However, only K‐nulling is required for MD estimation by STE, as applied in our experiments (see Methods). Considering that the final encoding is a result of a linear transformation **L**, Equation 14 can be written as

(15) $$M_{\mathrm{ij}}=\sum_{k,l}L_{\mathrm{ik}}L_{\mathrm{jl}}M\prime_{\mathrm{ij}}=0,\forall i,j\in \left(x,y,z\right),$$

where **M**′ is determined by the initial waveforms **g**^′^ = **L**^−1^**g**. Due to the symmetry, *M*_*ji*_ = *M*_*ij*_, Equations 14 and 15 require nulling of six independent elements of matrix **M**. For rank 2 b‐tensors, where $g_{i}^{\prime }\left(t\right)=0$ for any one component, we need to account for three conditions in Equations 14 and 15. For LTE, five components of **M**′ are always zero, thus only one condition in Equations 14 and 15 needs to be considered and M‐nulling in this case becomes trivial.^36^

For LTE, where the only nonzero component is $g_{i}^{\prime }\left(t\right)$, M‐nulling is fulfilled, for example, when

(16) $$g_{i}^{\prime }\left(\frac{\tau_{E}}{2}+t\right)=p_{i}\;g_{i}^{\prime }\left(t\right)\;\text{for}\;t<\frac{\tau_{E}}{2}\;\text{or}$$

(17) $$g_{i}^{\prime }\left(\frac{\tau_{E}}{2}+t\right)=p_{i}\;g_{i}^{\prime }\left(\frac{\tau_{E}}{2}-t\right)\;\text{for}\;t<\frac{\tau_{E}}{2},$$

where *p*_*i*_ =  ± 1, denotes polarity inversion. Equation 16 means that the waveform after refocusing pulse is an exact copy of the waveform before the refocusing pulse (with the same or inverted polarity). Equation 17 means that the waveform after refocusing pulse is a time‐reversed copy of the waveform before the refocusing pulse (with the same or inverted polarity). To generate LTE with moments nulled up to order 2, featuring four gradient lobes, we used Equation 16 in Welsh et al,^7^ which fulfils the time‐reversed symmetry of Equation 17 and thus nulls effects of concomitant gradients.

Considering a simple translational symmetry of Equation 16 for all gradient vector components in the case of rank 2 and rank 3 b‐tensors, when gradient waveforms from the first encoding block are repeated in the second encoding block with or without polarity inversion, M‐nulling can be achieved if at least one of the following conditions is met for ∀*i*,*j* ∈ (*x*,*y*,*z*):

(18) $$g_{i}^{\prime }\left(\frac{\tau_{E}}{2}+t\right)\;g_{j}^{\prime }\left(\frac{\tau_{E}}{2}+t\right)=g_{i}^{\prime }\left(t\right)\;g_{j}^{\prime }\left(t\right)\;\text{for}\;t<\frac{\tau_{E}}{2}\;\text{or}$$

(19) $$\int_{0}^{\tau_{E}/2}g_{i}^{\prime }\left(t\right)g_{j}^{\prime }\left(t\right)dt=0.$$

Equation 18 is always fulfilled for *i* = *j*, since $p_{i}^{2}=1$ for ∀*i*. It can also be fulfilled for any pair of components *i* ≠ *j*, if *p*_*i*_*p*_*j*_ = 1, ie, when the polarity is inverted or preserved for both *i*th and *j*th components. With our gradient moment nulling scheme, corresponding to the translational symmetry of Equation 16, Equation 18 and thus M‐nulling according to Equation 15 can be fulfilled for ∀*i*,*j* in the case of rank 2 b‐tensors, while for rank 3 b‐tensors, Equation 18 cannot be fulfilled for two cross terms with *i* ≠ *j*. However, since we have three free parameters for rotation and we need to null the remaining two cross terms, we can always find a rotation that can achieve K‐nulling according to Equation 11, given the translational symmetry of Equation 16.

### 2.4 Nulling effects of motion and concomitant gradients for b‐tensor encoding

**Figure 2:**
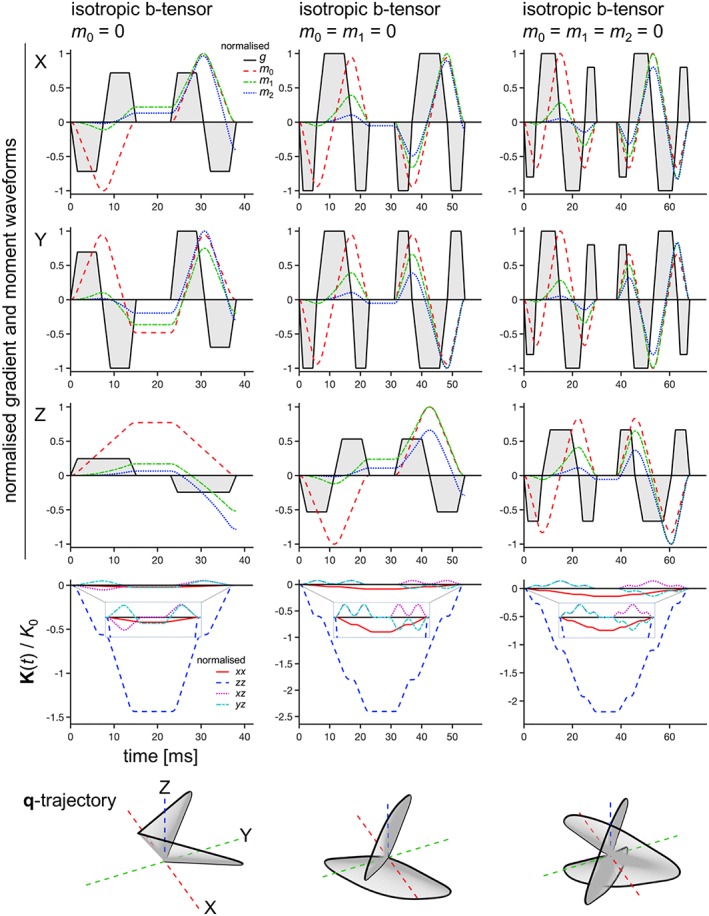
isotropic diffusion encoding with trapezoidal gradients, K‐nulling and motion nulling. Gradient moments are nulled up to 0th order (left column), 1st order (middle column) and 2nd order (right column). The first three rows show the time course of normalised effective gradient (black solid line), *m*_0_(*t*) (red dashed line), *m*_1_(*t*) (green dash‐dotted line) and *m*_2_(*t*) (blue dotted line) along x, y and z axes. Waveforms are normalised to the maximum magnitude across all three axes (for each column separately). The diffusion encoding durations (*τ*_E_) were 38, 54 and 68 ms (left to right). For up to 1st and 2nd order nulling, *b*_high_ was 0.45 ms/μm^2^, maximum gradient magnitude was 74 mT/m, and maximum slew rates were 59 and 54 T/m/s, respectively. The fourth line shows the time evolution of four independent and nonzero elements of matrix **K**. Elements of **K** are normalised by $K_{0}=\frac{\gamma }{2\pi }\tau_{E}\max\left|G_{C}\left(t\right)\right|$. The insets show zoomed regions of the original plots. The bottom line shows the q‐trajectory. Notice that the q‐vector is always parallel to one of two orthogonal q‐planes (grey surfaces)

**Figure 3:**
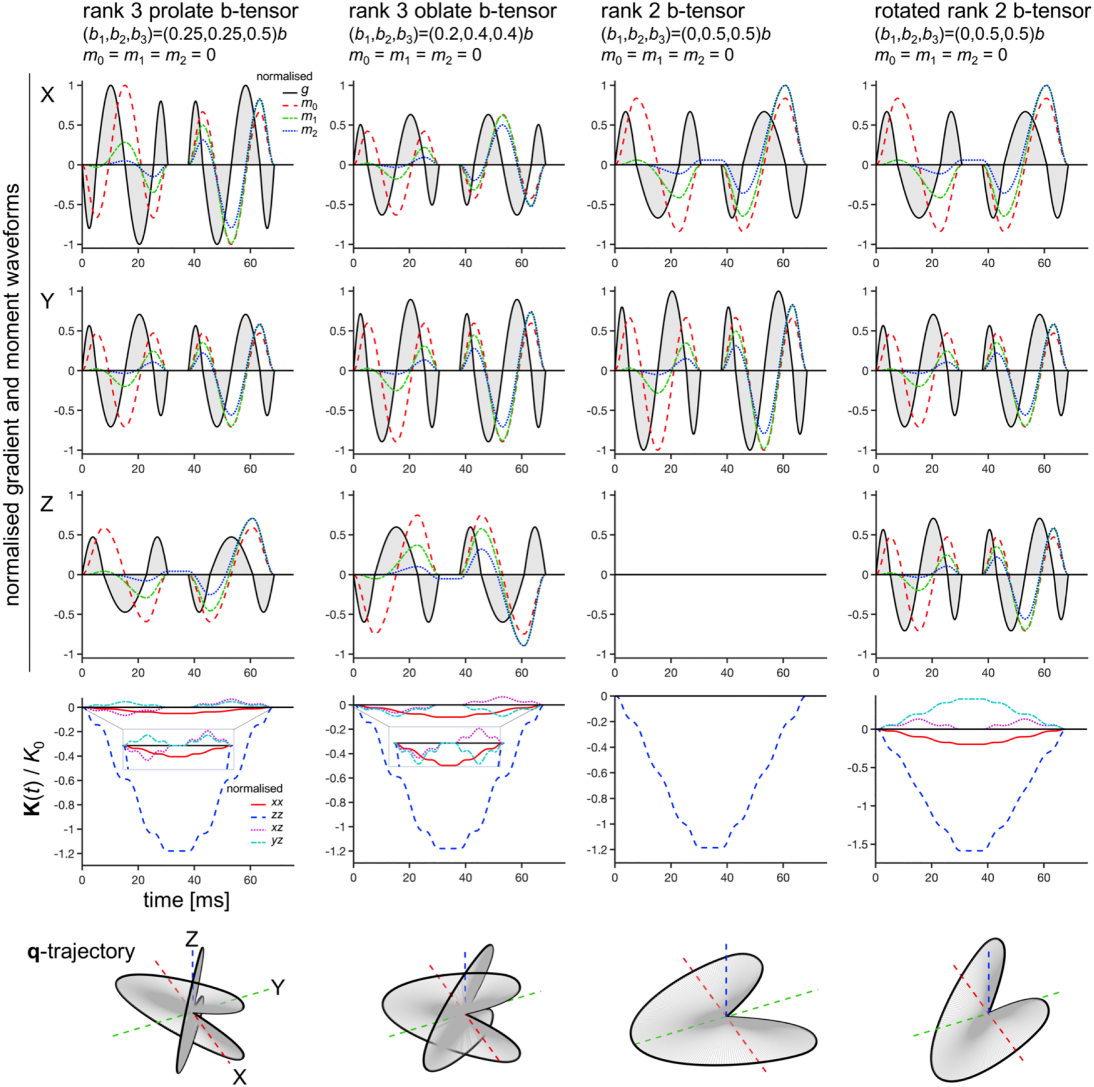
sinusoidal gradient waveforms for different shapes of b‐tensors with K‐nulling and motion nulling up to the 2nd gradient moment. Examples of different b‐tensor shapes include (left to right columns): Prolate with eigenvalues 0.25*b*, 0.25*b*, 0.5*b* (sorted in ascending order), oblate with eigenvalues 0.2*b*, 0.4*b*, 0.4*b* and rank 2 b‐tensors with eigenvalues 0, 0.5*b*, 0.5*b*. The rightmost column illustrates that for rank 2 b‐tensors, K‐nulling is invariant to rotation. This example features rotation by π/4 rad around x‐axis compared with the example shown in the third column. Note that for rank 3 b‐tensors, a specific rotation is required for K‐nulling. The same timing as in the case of acceleration nulling in Figure 2 is used here. Again, the first three rows show the time course of normalised effective gradient (black solid line), *m*_0_(*t*) (red dashed line), *m*_1_(*t*) (green dash‐dotted line) and *m*_2_(*t*) (blue dotted line) along x, y and z axes. For comparison, all waveforms are normalised to the global magnitudes (from all four columns). The fourth line shows the time evolution of four independent and nonzero elements of the **K** matrix. As in Figure 2, elements of **K** are normalised by $K_{0}=\frac{\gamma }{2\pi }\tau_{E}\max\left|G_{C}\left(t\right)\right|$. The insets show zoomed regions of the original plots. The bottom line shows the q‐trajectory

To generate rank 3 b‐tensors of arbitrary shape and size with gradient moments nulled up to an arbitrary order, we propose a composite SE‐encoding scheme outlined in Figure 1. Although Figure 1 features an example with motion nulling up to order 2, generalisation to arbitrary moments is straightforward. The general approach consists of two steps. First, the waveforms are initialised and then they are linearly transformed by scaling and rotation to achieve a desired b‐tensor and K‐nulling. The encoding is separated into two blocks of equal duration applied before and after the refocusing pulse. Two initial gradient waveforms are used as building blocks, which are repeated from the first to the second encoding block for different encoding axes. For these two initial waveforms, gradient moment nulling up to order 1 (M1‐waveform) and up to order 2 (M2‐waveform) can be achieved by using three and four gradient lobes of arbitrary durations, respectively. An uneven subdivision is illustrated in Figure 1 with the vertical dashed lines. However, in our experimental implementations, we used even subdivisions symmetric around the centre of encoding blocks, as featured in Figures 2 and 3.

To construct rank 2 b‐tensors, the M1‐waveform is initially applied along one axis and the M2‐waveform is applied along another axis, whereas for rank 3 b‐tensors, the M1‐waveform is applied along one axis and the M2‐waveform is applied along the other two axes. Note that in our pulse sequence design (Figure 1), “initialising” gradients along three orthogonal axes is required but not sufficient to generate rank 3 b‐tensors. If encoding is initialised along two orthogonal axes, rank 1 or rank 2 tensors can be generated, but not rank 3 tensors. A linear transformation will generally yield nonzero gradients along all orthogonal axes. To null gradient moments up to 2nd order, the M1‐waveform needs to preserve polarity in the second block (effective gradient changes polarity). To yield rank 3 b‐tensors, the M2‐waveform needs to change polarity in the second block along one of the axes. For rank 2 b‐tensors, the M2‐waveform can be set to zero along one of the axes, so that, eg, $g_{i}^{\prime }\left(t\right)=0$. However, M‐nulling requires fulfilling Equation 18 and thus *p*_*k*_*p*_*l*_ = 1 for *k*,*l* ≠ *i*. This means that for M‐nulled rank 2 b‐tensors, the polarity of M2‐waveform needs to be preserved in the second encoding block. The proposed encoding scheme can thus yield velocity and acceleration nulled rank 2 b‐tensor encoding with effects of concomitant gradients nulled independent of rotation. In this sense, rank 2 b‐tensors enjoy a special privilege. Motion and K‐nulling can be obtained for rank 3 b‐tensors of any shape, but in this case K‐nulling requires a specific rotation, which fulfils Equation 11. The last two observations are key features of our scheme. Note that when the initial encoding scheme, as shown in Figure 1, is linearly transformed to achieve the desired b‐tensor size and shape, the number of gradient lobes may increase along some axes. This is due to the interference between different gradient components, which occurs if subdivision for both M1‐ and M2‐waveforms is asymmetric relative to the centre of an encoding block. It is also important to note that rotation of gradients should always be the last step after the gradients have been rescaled for any desired b‐tensor shape.

Figure 2 illustrates an implementation of isotropic b‐tensors with gradient moments that are nulled up to 0th, 1st and 2nd order. While trapezoidal lobes generally allow achieving higher b‐values per gradient magnitude, alternative gradient waveforms may be used. An example featuring sinusoidal lobes is shown in Figure 3. Furthermore, arbitrary shapes of b‐tensors can be implemented, as illustrated in Figure 3, which shows examples of rank 3‐prolate, rank 3‐oblate and rank 2 b‐tensors.

## 3 METHODS

### 3.1 Generating spherical tensor encoding waveforms

Gradient waveforms for STE with K‐nulling and moment nulling up to the 2nd order were generated according to the principles outlined in the Theory section (see Figure 1). For our proof‐of‐concept experiments, we used trapezoidal gradient waveforms. Such waveforms are known for their high b‐value efficiency, ease of specification, and are routinely employed in both imaging and diffusion‐weighting.

Our STE gradient waveforms with moments nulled up to order *n* = 0, 1, 2 (shown in Figure 2) were generated based on the following steps:

- Two gradient waveforms with *n* + 1 (*waveform 1*) and *n* + 2 (*waveform 2*) trapezoidal lobes were generated. The duration of these waveforms was chosen to fit on each side of the refocusing pulse while also allowing for gradients to be turned off during the interval surrounding the refocusing pulse (see timing in Figure 2). Ramp times of 1.6 ms were used.
- Copies of *waveform 1* were used with opposite polarities on each side of the refocusing pulse along the x‐axis (axis 1 in Figure 1). Copies of *waveform 2* were used with opposite polarities on each side of the refocusing pulse along the y‐axis (axis 2 in Figure 1) and with equal polarities on each side of the refocusing pulse along the z‐axis (axis 3 in Figure 1).
- The resulting waveforms were scaled to yield isotropic b‐tensors.
- Finally, the waveforms were rotated to achieve K‐nulling.

Matlab code to generate gradient waveforms for tensor‐valued diffusion encoding with gradient moment nulling up to 2nd order as well as nulling effects of concomitant gradients are available upon request.

### 3.2 In vivo experiments

Cardiac DWI data were acquired in healthy volunteers (*N* = 5). The study was conducted in accordance with the Declaration of Helsinki and was approved by the UK National Research Ethics Service (18/YH/0168). All patients provided written informed consent. A Prisma 3 T MRI scanner with maximum gradient magnitude of 80 mT/m and maximum slew rate of 200 T/m/s (Siemens Healthineers, Erlangen, Germany) was used. Balanced steady‐state free precession cine data were acquired in 3‐planes and used to define the time from R peak to maximum systole. The trigger delay was defined as ~ 30% of maximum systole.

To evaluate the measurement of MD using the proposed approach, we acquired data with STE waveforms and gradient moment nulling up to 1st and 2nd moments (M1STE, M2STE). These were compared with current methods including STE without moment nulling (M0STE) and LTE with gradient moment nulling up to 0th, 1st and 2nd moments (M0LTE, M1LTE, M2LTE). A prototype single‐shot SE EPI sequence^30^ was used to execute M1STE, M2STE (as shown in Figure 2). A radiofrequency ZOOM‐IT (Siemens Healthineers, Erlangen, Germany) excitation and cardiac triggering were used.^37^ For the M0STE case, we used a waveform similar to those proposed in Sjolund et al^29^ (M0STE), which yields a factor of 1.2‐fold higher b‐values at equal gradient magnitudes compared with the proposed waveform shown in the left column of Figure 2. Corresponding M0LTE, M1LTE and M2LTE data were acquired using monopolar,^38^ bipolar and asymmetric bipolar^6^, ^7^ gradient waveforms (not shown). The imaging parameters were: TR = 5 cardiac cycles (ie, 5 s based on a heart rate of 60 beats per minute), field‐of‐view = 320 × 118 mm^2^, in‐plane resolution = 3.0 × 3.0 mm^2^, slice thickness = 8 mm, number of slices = 5, partial Fourier = 6/8, no parallel imaging, bandwidth = 1494 Hz/pixel, *b*_low_ = 0.1 ms/μm^2^ and *b*_high_ = 0.45 ms/μm^2^. Data were acquired during free‐breathing without respiratory gating. A nonzero low b‐value was used to suppress the effects of myocardial perfusion.^39^ Crusher gradients were used because the diffusion encoding gradients are self‐balanced, ie, they have a zeroth moment of zero on either side of the refocusing pulse. For all STE waveforms, the minimum echo time was used. For M0STE, M1STE and M2STE, the TE was 60, 76 and 90 ms, respectively, and the corresponding encoding durations were 38, 54 and 68 ms, respectively. Gradients were turned off for 8 ms at the time of RF refocusing. Despite the higher nominal maximum slew rate available, slew rates were progressively reduced in order to avoid exceeding scanner‐defined peripheral nerve stimulation (PNS) limits.^40^ The echo time in LTE was matched to that of the STE acquisitions. In addition, LTE data with minimum echo times (M0LTEmin, M1LTEmin and M2LTEmin) were acquired with TE = 51, 72 and 80 ms, respectively (*N* = 4). In order to improve the accuracy of our reference data, each scan was acquired with *N*_acq_ = 48 acquisitions, including two b‐values, one or three orthogonal diffusion‐weighted (DW) directions, and 24 or eight repetitions (*N*_rep_) for STE or LTE, respectively. The total acquisition time per scan excluding scanner preparation was 4 minutes. The specific ordering of b‐values, DW directions and *N*_rep_ is given in Table 1.

**Table 1:** Ordering of scans up to *N*_acq_ = 48. Total scan time, as determined by *N*_acq_, was consistent between spherical tensor encoding (STE) and linear tensor encoding (LTE). *N*_rep_ indicates the number of repetitions with the same b‐value and diffusion‐weighted (DW) direction

	Ordering of scans								
STE	*N*_acq_	1	2	3	4	5	6	…	48
	*N*_rep_	1	1	2	2	3	3	…	24
	*b* (ms/μm^2^)	0.1	0.45	0.1	0.45	0.1	0.45	…	0.45
	DW	Iso	Iso	Iso	Iso	Iso	Iso	…	Iso
LTE	*N*_acq_	1	2	3	4	5	6	…	48
	*N*_rep_	1	1	1	1	1	1	…	8
	*b* (ms/μm^2^)	0.1	0.45	0.1	0.45	0.1	0.45	…	0.45
	DW	X	X	Y	Y	Z	Z	…	Z

### 3.3 Data analysis

Diffusion‐weighted images were affinely registered in 2D within‐subject using Elastix^41^ based on a reference image calculated from the mean of the unregistered *b* = 0.1 and 0.45 ms/μm^2^ M2STE and M2LTE data. The left ventricular (LV) myocardium was segmented manually based on this reference image. SNR of the trace signal was measured in LV myocardium in data acquired with a *b*‐value of 0.45 ms/μm^2^ according to the method of Dietrich et al,^42^ ie, by voxelwise division of the mean and standard deviation (SD) signal intensities in the trace‐weighted images across *N*_rep_, followed by averaging across voxels and subjects. To match acquisition times, trace‐weighted images were calculated from the average of consecutive blocks of three acquisitions in both STE and LTE cases.

The MD was calculated as

(20) $$\mathrm{MD}=\frac{1}{N_{\mathrm{acq}}/2}\sum_{i=1}^{N_{\mathrm{acq}}/2}\frac{1}{\left(b_{\text{high}}-b_{\mathrm{low}}\right)\;}\;\ln\left[\frac{S_{i}\left(b_{\mathrm{low}}\right)}{S_{i}\left(b_{\text{high}}\right)}\right]\;,$$

where *S*_*i*_(*b*_low_) and *S*_*i*_(*b*_high_) are signal intensities at low and high b‐values at the *i*th acquisition, respectively. The MD was averaged across the LV myocardium in a single mid‐myocardial slice, and its mean and SD reported across volunteers.

To minimise sampling bias associated with small *N*_acq_, the MD maps were permuted in 24 different combinations, in blocks of *N*_acq_, using a circular sliding window. Regional heterogeneity was determined by the within‐subject SD of the MD, averaged across the LV myocardium in a single mid‐myocardial slice, and across permutations and subjects.

### 3.4 Phantom experiments

To complement the in vivo experiments, the same data acquisition and analysis were performed in (a) an isotropic phosphate‐buffered saline (PBS)‐based 1% agarose gel cylinder phantom, and (b) an unfixed ex vivo pig heart (*N* = 1) obtained from the local butcher, which was embedded in the same gel. In both experiments, a simulated electrocardiogram with a rate of 60 beats per minute was used.

## 4 EXPERIMENTAL RESULTS

**Figure 4:**
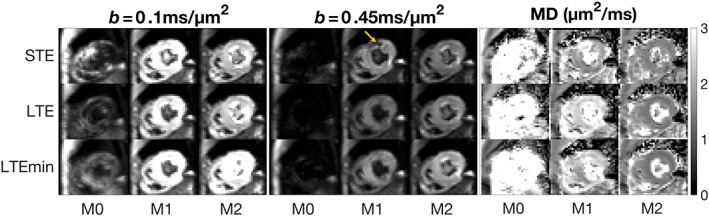
representative mid‐myocardial diffusion‐weighted (DW) images and mean diffusivity (MD). Data were acquired with M0, M1 and M2‐nulling, and spherical tensor encoding (STE), linear tensor encoding (LTE), and LTE with minimised echo time (LTEmin) and with *N*_acq_ = 48. M2‐nulling yielded consistent data free of local signal dropouts (arrow) arising from incomplete motion compensation

**Figure 5:**
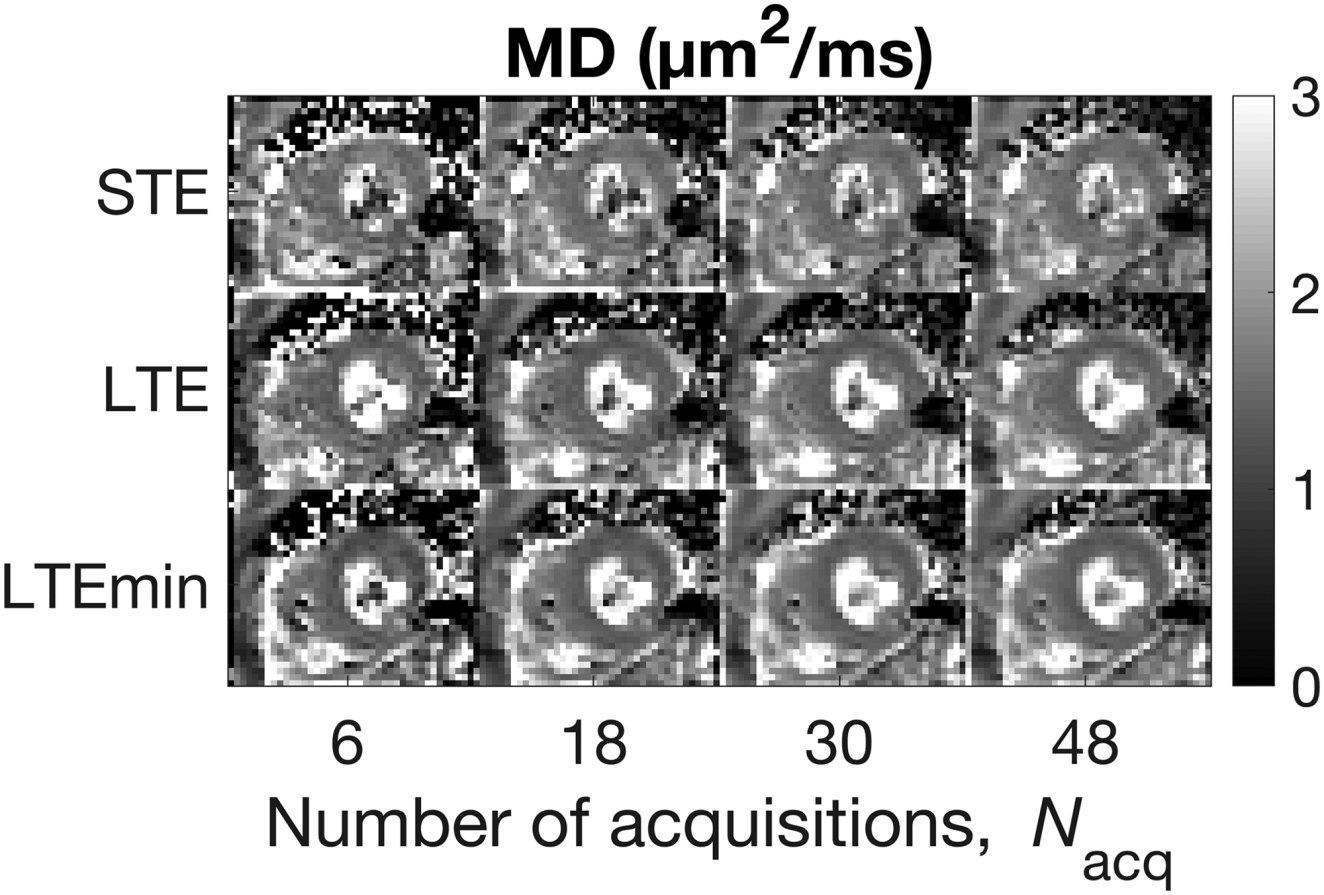
Representative M2‐nulled mid‐myocardial mean diffusivity (MD) maps reconstructed from number of acquisitions, *N*_acq_. The SNR for *b* = 0.45 ms/μm^2^ data in the left ventricular (LV) myocardium averaged across subjects was 15.9, 14.7 and 20.7 for spherical tensor encoding (STE), linear tensor encoding (LTE) and LTE with minimised echo time (LTEmin), respectively. For clarity, data are shown for subsets with different *N*_acq_

Representative mid‐myocardial images at *b* = 0.1 and 0.45 ms/μm^2^ are shown alongside MD maps (Figure 4). Marked signal loss was observed throughout the myocardium in the M0‐nulled data, precluding image registration and artificially enhancing MD. The M1‐nulled and M2‐nulled data were more regionally homogeneous, although local signal dropouts were observed, particularly in the *b* = 0.45 ms/μm^2^ M1‐nulled images. MD maps reconstructed from the M2‐nulled data using 6 ≤ *N*_acq_ ≤ 48 in one subject illustrate the effect of repetitions upon the data quality (Figure 5).

**Figure 6:**
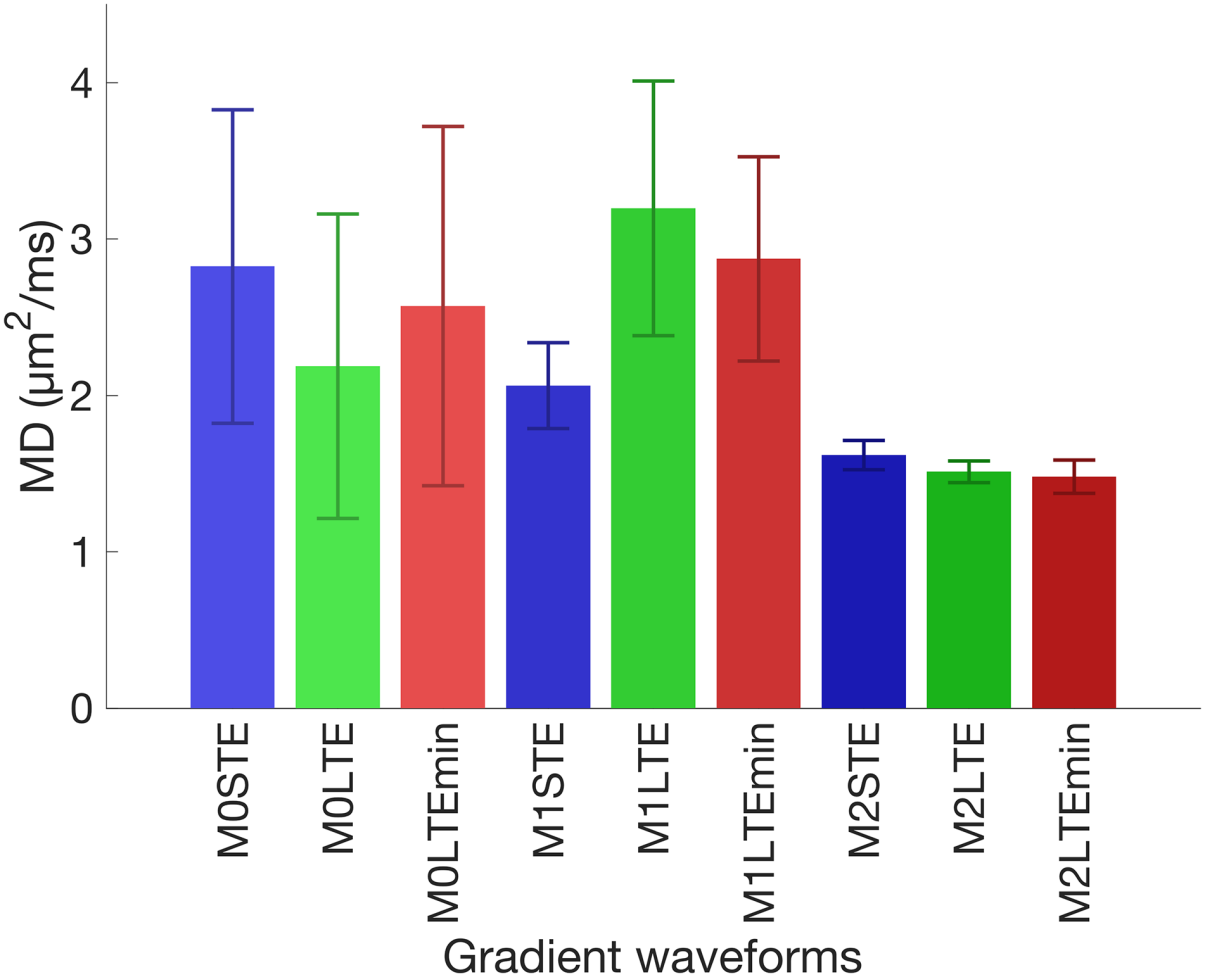
Mean diffusivity (MD) across the left ventricular (LV) myocardium in mid‐myocardial slice with *N*_acq_ = 48 (mean ± standard deviation [SD] across subjects). M2STE exhibited 7% (*P* = 0.029) and 9% (*P* = 0.075) higher MD than M2LTE and M2LTEmin, respectively (paired one‐tailed t‐test). These differences in MD may be attributed to different sensitivities of linear tensor encoding (LTE) and spherical tensor encoding (STE) gradient waveforms to restricted diffusion

The MD averaged across the myocardium (mean ± SD across subjects) was highest in the M0‐nulled sequences, lowest in the M2‐nulled sequences, and intermediate in the M1‐nulled sequences (Figure 6). This indicates an artificial loss of signal for nonmotion‐compensated waveform designs. In the M1‐nulled and M2‐nulled data, MD was consistent across all values of *N*_acq_. Within M2‐nulled sequences, M2STE exhibited 7% (*P* = 0.029) higher MD than M2LTE and 9% (*P* = 0.075) higher MD than M2LTEmin (paired one‐tailed t‐test).

**Figure 7:**
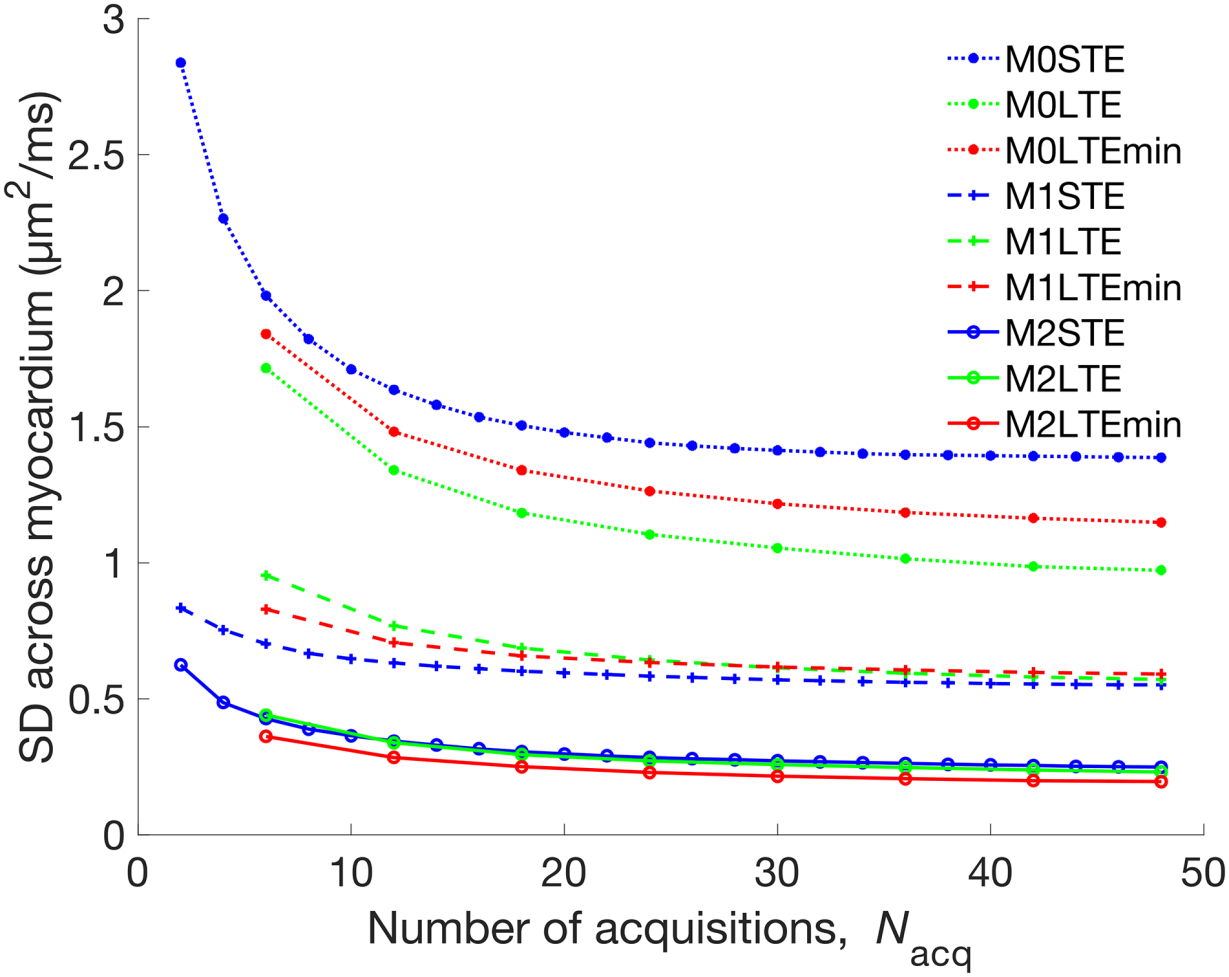
Regional heterogeneity over left ventricular (LV) myocardium as measured by the standard deviation (SD) of mean diffusivity (MD) across voxels and averaged across subjects. The SD decreased as the order of motion nulling increased up to M2‐nulling. The differences in SD between spherical tensor encoding (STE), linear tensor encoding (LTE) and LTE with minimised echo time (LTEmin) within each order of motion nulling were much smaller than those across different orders of motion nulling

The presence of local signal dropouts was also reflected in the low, medium and high regional heterogeneity seen in the M2‐nulled, M1‐nulled and M0‐nulled sequences (Figure 7). Regional heterogeneity was found to decrease as the order of motion nulling used increased. It also decreased with increasing *N*_acq_ (Table 2). The M2‐nulled waveforms yielded the lowest heterogeneity. Incomplete motion compensation in the myocardium resulted in localised signal loss using the M1‐nulled waveforms. This was apparent in both LTE and STE data, and was consistent with previous work.^7^ The signal dropouts with the M0‐nulled sequences were consistent with known sensitivity to the cardiac motion of nonmotion‐compensated LTE sequences. The artefactual signal loss due to uncompensated motion was also reflected in the MD as a function of *N*_acq_. We observed that all M0‐nulled sequences yielded nonphysiological MD values near or exceeding that of water^43^ at 37°C, ie, 2.96 μm^2^/ms.

**Table 2:** Standard deviation (μm^2^/ms) of mean diffusivity (MD) across voxels in the left ventricular (LV) myocardium (mean ± standard deviation [SD] across subjects). SDs were lowest in the M2‐nulled data. Based on 6 ≤ *N*_acq_ ≤ 48, SD of M2LTE and M2LTEmin were on average 3% and 19% lower, respectively, compared with that of M2STE

*N*_acq_	6	12	18	24	48
M0STE	1.98 ± 0.20	1.64 ± 0.21	1.50 ± 0.23	1.44 ± 0.23	1.39 ± 0.25
M0LTE	1.71 ± 0.13	1.34 ± 0.12	1.18 ± 0.11	1.10 ± 0.11	0.97 ± 0.12
M0LTEmin	1.84 ± 0.14	1.48 ± 0.13	1.34 ± 0.14	1.26 ± 0.14	1.15 ± 0.14
M1STE	0.70 ± 0.23	0.63 ± 0.21	0.60 ± 0.20	0.58 ± 0.20	0.55 ± 0.22
M1LTE	0.95 ± 0.24	0.77 ± 0.24	0.69 ± 0.24	0.64 ± 0.25	0.57 ± 0.26
M1LTEmin	0.83 ± 0.20	0.71 ± 0.16	0.66 ± 0.14	0.63 ± 0.14	0.59 ± 0.13
M2STE	0.43 ± 0.21	0.34 ± 0.24	0.31 ± 0.27	0.28 ± 0.28	0.25 ± 0.27
M2LTE	0.44 ± 0.18	0.34 ± 0.19	0.30 ± 0.20	0.27 ± 0.20	0.23 ± 0.22
M2LTEmin	0.36 ± 0.18	0.28 ± 0.13	0.25 ± 0.11	0.23 ± 0.09	0.20 ± 0.08

**Figure 8:**
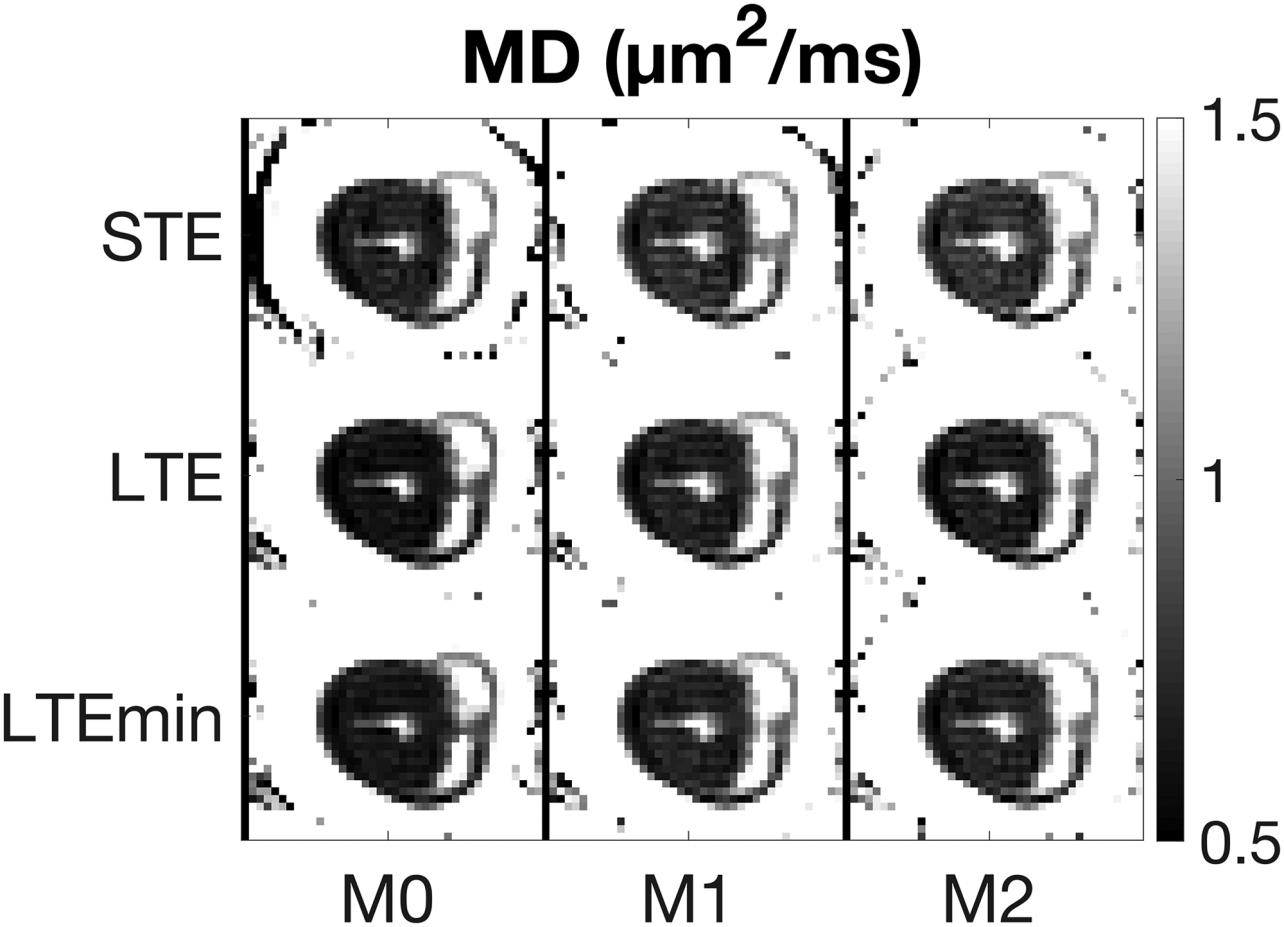
Representative mid‐myocardial mean diffusivity (MD) in static ex vivo pig heart embedded in gel. Data were acquired with M0, M1 and M2‐nulling, and spherical tensor encoding (STE), linear tensor encoding (LTE), and LTE with minimised echo time (LTEmin) with *N*_acq_ = 48. No artefacts arising from uncompensated motion were observed

**Figure 9:**
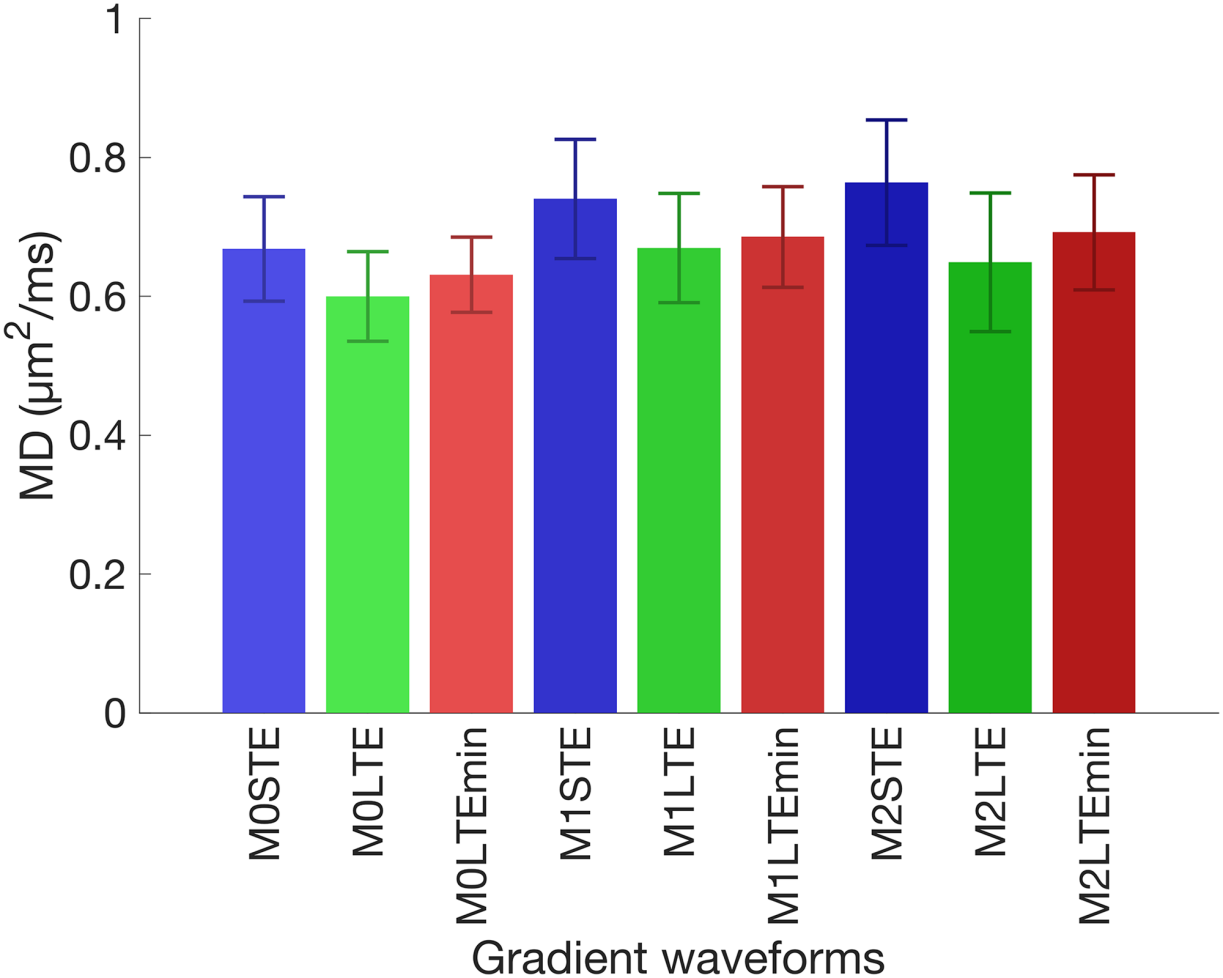
Voxel‐average mean diffusivity (MD) across the static ex vivo pig heart in mid‐myocardial slice with *N*_acq_ = 48 (mean standard deviation [SD] across voxels). MD values were 18% and 10% higher for M2STE compared with M2LTE and M2LTEmin, respectively. Similar trends of average MD values were observed for M0STE and M1STE compared with the linear tensor encoding (LTE). The observed differences in MD could be attributed to different sensitivities of spherical tensor encoding (STE) and LTE waveforms to restricted diffusion

In the phantom experiments, MD in the isotropic gel was uniform across the different sequences used; MD_gel_ = 2.07 ± 0.01 (μm^2^/ms; mean ± SD across sequences) with a coefficient of variation across sequences of 0.7%. Representative mid‐myocardial images of the ex vivo pig heart are shown, with no signal dropouts associated with uncompensated motion (Figure 8). The MD in the myocardium was observed to generally increase with order of motion compensation. Within the same order of motion compensation, the MD was highest in the STE acquisitions and lowest in the LTE acquisitions (Figure 9).

## 5 DISCUSSION

Here we present a comprehensive analysis of motion nulling, applicable to b‐tensors of arbitrary shapes, while avoiding the confounding effects of concomitant gradients. Proof‐of‐concept experiments with acceleration‐compensated isotropic diffusion encoding were performed in vivo in the human heart.

Tensor‐valued encoding has in recent years gained a lot of interest due to its supreme specificity for studying microstructure.^19^, ^26^, ^28^ While optimisations of gradient waveforms for tensor‐valued encoding can deliver formidable efficiency improvements,^29^, ^30^ motion‐compensated designs have, however, not yet been thoroughly explored in this context. The potential for fast in vivo quantification of MD with higher order motion‐compensated isotropic encoding represents an introductory application of tensor‐valued encoding in organs with significant bulk motion, such as the heart.

Isotropic diffusion weighting was introduced early on,^17^ but only velocity compensation has been implemented in isotropic weighting.^16^ Motion compensation up to the 2nd gradient moment and higher has only been implemented for the conventional LTE.^6^, ^7^, ^9^, ^13^ Optimisations of diffusion encoding in the context of SE sequences take advantage of waveforms that are asymmetric around the refocusing pulse, for both LTE^13^ and STE.^29^ Such waveforms may cause unwanted signal attenuation away from the isocentre due to concomitant field gradients leading to erroneous quantification and reduced image quality.^14^ The effects of concomitant gradients are often avoided by employing less efficient symmetric encoding waveforms, such as in the case of isotropic weighting by Mori and van Zijl,^17^ or not considered, such as in the velocity‐compensated design of Wong et al.^16^ The M2‐nulled LTE scheme by Alliota et al^13^ can minimise echo times by employing asymmetric gradients, but unfortunately leads to nonzero concomitant gradients according to Equation 12.^14^ By contrast, LTE with gradient moments nulled up to order 2 or 3, as suggested by Welsh et al,^7^ fulfils Equation 17 and thus nulls the effects of concomitant gradients independent of rotation.

In vivo, the MD in M2STE was higher than that of M2LTE. These observations suggest that the differences in MD measured in vivo could be due to time‐dependent diffusion effects and/or microscopic diffusion anisotropy. Time‐dependent diffusion effects can be studied by considering the analytical model of diffusion restricted in impermeable cylinders^44^, ^45^ and are expected because the M2LTE waveform has more encoding power at lower frequencies (longer times) compared with the M2STE waveform.^45^ Because apparent diffusivity is reduced at longer times due to restrictions, the increased sensitivity of the M2LTE waveform to diffusion at longer times yields lower signal attenuation compared with the M2STE waveform. This is consistent with our previous work examining time‐dependent diffusion in ex vivo mouse heart, where we showed that MD, as measured using LTE with pulsed and oscillating gradients, decreased as effective diffusion time increased.^46^ To preclude the potential effects of different motion sensitivities between LTE and STE on the measured MD, the experiments were repeated ex vivo. We observed a similar higher MD in M2STE compared with M2LTE in ex vivo pig heart, while uniform MD was observed across sequences in the isotropic gel phantom. This was consistent with the hypothesis that the MD variation between sequences could stem from time‐dependent diffusion effects and/or microscopic diffusion anisotropy.

Differences in MD between STE and LTE could also result from anisotropic Gaussian diffusion. It is known that LTE methods tend to underestimate the trace of the diffusion tensor in the presence of microscopic anisotropy^24^ and macroscopic dispersion.^16^ This may occur when relatively high b‐values are used, sensing multiexponential signal attenuation.^16^ The relative contributions of the time‐dependent diffusion effect and the diffusion anisotropy, which can both cause higher MD in STE, depend on the size of restrictions. Our computations (data not shown) suggest that for typical cardiomyocytes with diameters of 19 microns,^47^ the time‐dependent diffusion effect would be the primary cause for the higher MD observed in our experiments with STE using relatively low b‐values.

MD across subjects was stable across *N*_acq_ in the M2‐nulled sequences. However, reducing *N*_acq_ resulted in increased regional heterogeneity. Therefore, while a nominal scan time of 10 seconds is achievable using M2STE, its applicability will be governed by the anticipated MD contrast between healthy and diseased myocardium and the underlying signal characteristics. In myocardial infarction, for example, an increase in MD of 12% was detected in patient infarcts relative to remote myocardium.^48^ In ex vivo DTI of pig hearts, the MD was 43%^49^ and 51% higher in infarcts relative to remote myocardium.^50^ Such marked differences, as observed in the ex vivo data, could justify the choice of *N*_acq_ = 2. By contrast, the in vivo patient data suggest that *N*_acq_ ≥ 6 and acquisition times of at least 30 seconds may be needed for robust MD measurements. One consequence of free‐breathing approaches is sensitivity to through‐slice motion, which is often mitigated by averaging. Through‐slice motion may be a reason, besides limited SNR, for the increase in regional heterogeneity as *N*_acq_ decreases down to 1 in M2STE. The benefit of reducing *N*_acq_ to 1 with a nominal scan time of 10 seconds, however, is that it permits breath‐holding, which may improve the data quality by avoiding respiratory motion.

The main limitation to our approach is the requirement for timing symmetry of gradient waveforms about the refocusing pulse. This can be suboptimal in terms of gradient waveform efficiency and lead to longer TE where an EPI readout is used.^14^ In this context, we found that the M2STE had 8% higher SNR than M2LTE, but 23% lower SNR than LTEmin. If averaging is needed, then it becomes more efficient to employ LTE waveforms over STE waveforms due to the shorter minimum TE of LTE. Optimising encoding waveforms for SNR and b‐value is crucial for successful applications, and numerical optimisation methods^13^, ^29^ have the potential to provide solutions to maximise gradient waveform efficiency, and thereby shorten TE, while also nulling the effects of both motion and concomitant gradients.

As the number of lobes in the gradient waveforms increases with progressively higher orders of gradient moment nulling, so does the slew rate have a greater impact on the gradient waveform efficiency. The higher gradient slew rates required for gradient moment nulling pose a risk of exceeding PNS limits. Methods to reduce PNS, and the peak PNS in particular, include reducing slew rates and the duration of gradient ramps and potentially using nontrapezoidal waveforms. However, these are likely to reduce the gradient waveform efficiency. Where possible, the considerable contribution of the EPI readout to PNS can be reduced, eg, by reducing the imaging resolution. PNS could also be modelled^51^ and incorporated into a gradient waveform optimisation procedure.

Another consideration is the extent to which *m*_1_ and *m*_2_ moments need to be nulled. Based on simulations, Middione et al reported that a negligible increase in the apparent diffusion coefficient is observed when the residual *m*_1_ and *m*_2_ are of the order of 10^−4^ mT/m·s^2^ and 10^−5^ mT/m·s^3^, respectively.^52^ While the diffusion waveforms used herein had negligible residual *m*_1_ and *m*_2_ at the level of numerical precision, the use of crushers would have resulted in residual *m*_1_ and *m*_2_ of the order of 10^−4^ mT/m·s^2^ and 10^−4^ mT/m·s^3^, respectively, along the crusher axes. This may potentially have led to nonnegligible signal attenuation, although the sensitivity of in vivo measurements to residual moments remains to be investigated. Further reduction of residual moments may be achievable by incorporating crushers into the overall gradient waveform design, with associated improvements in robustness to higher velocities and acceleration.

In SE EPI, B_1_ inhomogeneity primarily leads to loss in SNR due to imperfect excitation and refocusing; it may also degrade fat saturation that relies on inversion pulses. Neither of these were observed to be major issues within the myocardium at 3 T. B_1_ homogeneity may be improved by the use of composite or adiabatic RF pulses, although the benefit may be more apparent at higher field strengths.

In summary, we have presented a gradient waveform design for tensor‐valued diffusion encoding with gradient moment nulling up to an arbitrary order. We have shown that the proposed waveform design can yield rank 2 b‐tensors with concomitant gradient effects nulled independent of waveform rotation. For rank 3 b‐tensors, eg, for STE, a specific rotation is required to null concomitant gradient effects. This condition does not prevent estimation of microscopic fractional anisotropy based on LTE and STE data,^24^, ^25^ but may limit an assessment of further microstructural information, which requires directional averaging of data acquired with rank 3 anisotropic b‐tensors.^26^, ^27^, ^28^ We compared the performance of current M2LTE with proposed M2STE for cardiac application and found that the results were comparable insofar as the stability of MD measurements. One potential benefit of M2STE is the possibility of reducing the number of required repetitions to a single average, consequently shortening scan times, which may permit breath‐holding and result in improved data consistency. Further work is needed to investigate the prospect of single breath‐hold fast mapping of MD in relevant patient cohorts. Reducing acquisition times may also encourage wider clinical application of cardiac diffusion MRI. Our future work will focus on improving gradient waveform efficiency for different applications. The suggested STE is nonapplication‐specific and can be applied in other organs such as the kidneys,^53^ prostate^54^ and liver, where motion can be problematic. Finally, the proposed encoding design could facilitate novel technical developments such as isolating the signal contribution of perfusion^55^ and/or acceleration, and investigating multidimensional diffusion encoding for improving the specificity of microstructure characterisation with diffusion MRI, as demonstrated in imaging of tumours.^56^, ^57^

## CONFLICT OF INTEREST

Related to this manuscript, Random Walk Imaging filed a Swedish patent application 1950507–2 (dated 26 April 2019) with SL, FS, MN and IT as inventors. Random Walk Imaging also hold the related patents PCT/SE2015/050156 and WO 2015/119569 with SL and MN as coinventors.

## FUNDING INFORMATION

This work was supported by the British Heart Foundation, UK (SI/14/1/30718), National Institutes of Health (P41EB015902) and Random Walk Imaging.
